# Mass Spectrometry Imaging Disclosed Spatial Distribution of Defense-Related Metabolites in *Triticum* spp.

**DOI:** 10.3390/metabo12010048

**Published:** 2022-01-07

**Authors:** Laura Righetti, Sven Gottwald, Sara Tortorella, Bernhard Spengler, Dhaka Ram Bhandari

**Affiliations:** 1 Institute of Inorganic and Analytical Chemistry, Justus Liebig University Giessen, Heinrich-Buff-Ring 17, 35392 Giessen, Germany; sv.gottwald@t-online.de (S.G.); bernhard.spengler@anorg.chemie.uni-giessen.de (B.S.); 2 Food and Drug Department, University of Parma, Viale delle Scienze 17/A, 43124 Parma, Italy; 3 Molecular Horizon srl, Via Montelino 30, Bettona, 06084 Perugia, Italy; sara@molhorizon.it; 4 Gandaki Prvince Academy of Science and Technology, Pokhara 33700, Nepal

**Keywords:** mass spectrometry imaging, plant defense metabolites, mycotoxins, fungal infection, wheat

## Abstract

Fusarium Head Blight is the most common fungal disease that strongly affects *Triticum* spp., reducing crop yield and leading to the accumulation of toxic metabolites. Several studies have investigated the plant metabolic response to counteract mycotoxins accumulation. However, information on the precise location where the defense mechanism is taking place is scarce. Therefore, this study aimed to investigate the specific tissue distribution of defense metabolites in two *Triticum* species and use this information to postulate on the metabolites’ functional role, unlocking the “location-to-function” paradigm. To address this challenge, transversal cross-sections were obtained from the middle of the grains. They were analyzed using an atmospheric-pressure (AP) SMALDI MSI source (AP-SMALDI5 AF, TransMIT GmbH, Giessen, Germany) coupled to a Q Exactive HF (Thermo Fisher Scientific GmbH, Bremen, Germany) orbital trapping mass spectrometer. Our result revealed the capability of (AP)-SMALDI MSI instrumentation to finely investigate the spatial distribution of wheat defense metabolites, such as hydroxycinnamic acid amides, oxylipins, linoleic and α-linoleic acids, galactolipids, and glycerolipids.

## 1. Introduction

Fusarium Head Blight (FHB), the most common fungal disease that strongly affects *Triticum* spp., causes grain yield losses and an accumulation of toxic secondary metabolites called mycotoxins [1,2]. The impact of these highly toxic fungal metabolites in the agro-food chain is extremely relevant, with up to 80% of food crops being contaminated worldwide [3] with deoxynivalenol (DON) and zearalenone present in most of the samples and at the highest concentrations. In addition, the annual loss caused by this contaminant in farming and aquaculture industries is estimated to be about 1 billion metric tons of food [4,5]. This scenario is expected to be exacerbated by climate change, due to the changing condition of temperature and humidity, favorable for fungal growth [6,7]. To tackle this challenge, the use of resistant cultivars able to mitigate the mycotoxins accumulation is considered the most promising approach [8,9]. This strategy requires a deep comprehension of the defense mechanisms activated in plants after fungal invasion for developing possible strategies to facilitate precise breeding. However, the host resistance mechanisms are still poorly understood. In the past decade, metabolomic strategies have compared mock vs. *Fusarium*-inoculated samples to decipher the chemical defense that cereals employ to counteract *Fusarium* spp. [10,11,12,13,14]. Both polar metabolites [13,15,16,17] and lipids [12,18] have been found to be modulated in plants following this biotic stressor. This approach has extended our knowledge, revealing some biological pathways by which the plant interacts against fungal attacks.

Nevertheless, the primary constraint is related to the complexity of the plant metabolome. On the one hand, these metabolites cannot be isolated by a single extraction procedure, and consequently, a single analytical method cannot take the entire picture. On the other hand, metabolomics approaches miss spatial information due to the extraction process. Localizing where the defense mechanism is taking place is essential to postulate on the biochemical process and assign each metabolite a specific role. The need to unlock the “location-to-function” paradigm in plant science has also been discussed by the international Plant Cell Atlas initiative [19,20].

In recent years, visualization has become an essential feature in plant science, enabling us to study the metabolites’ distributions within a tissue. In this regard, mass spectrometry imaging (MSI) has become a powerful tool capable of achieving spatial distributions and chemical specificity by enabling unprecedented details of metabolic biology to be uncovered. However, the scientific literature mainly focuses on describing the alteration of physiological mechanisms, and limited information is available regarding the distribution of mycotoxins and defense-related metabolites, not taking full advantage of modern analytical tools, such as those made available by MSI. Recently, few studies [21,22,23] have visualized *in situ* the chemical response of cereals to FHB, revealing the spatial distribution of carnitine and tetra-hexose [21], antimicrobial flavonoid glycosides [23], chlorophyll derivates [23], terpenoids, and hydroxycinnamic acid amide [22]. However, the complex and integrated network of events that cereals can orchestrate to resist *Fusarium* spp. includes a broader range of metabolites. For instance, upon pathogen infection, various crops accumulate essential signaling lipids, including glycerophospholipids and galactolipids. Furthermore, the growing literature reports the central role of oxylipins as key signaling compounds involved in the plant-pathogen cross-talk [12,18,24,25].

The present study aims to visualize the tissue distribution of metabolites and lipids with a role in mediating the wheat response to *Fusarium* and DON accumulation. To address this challenge, transversal cross-sections of two *Triticum* spp. were obtained from the middle of the grains and were analyzed using an atmospheric-pressure (AP) SMALDI MSI source (AP-SMALDI5 AF, TransMIT GmbH, Giessen, Germany) coupled to a Q Exactive HF (Thermo Fisher Scientific GmbH, Bremen, Germany) orbital trapping mass spectrometer. The metabolites previously reported in the literature, including hydroxycinnamic acids, oxylipins, linoleic and α-linoleic acids, galactolipids, and diacylglycerols, were imaged in infected vs. non-infected common and durum wheat kernels.

## 2. Results and Discussion

The metabolites previously reported in the literature to have a role in modulating wheat response [10,12,15] were imaged in infected vs. non-infected wheat kernels. Their spatial distribution was studied using a transversal cross-section made from the middle of the grains.

Common and durum wheat samples were analyzed one year from each other and using two instruments. Therefore, the consistent results strengthened the hypothesis on the role of the identified metabolites in the *Fusarium* pathogenicity and indicated the high reproducibility of the measurements.

To reduce the measurement time, the pixel size was not set to the smallest possible value (such as 3 µm as demonstrated by Rompp et al., [26]). The selected pixel resolution of 20 µm was found to be well-adapted to the tissue and the histological question.

**Figure 1:**
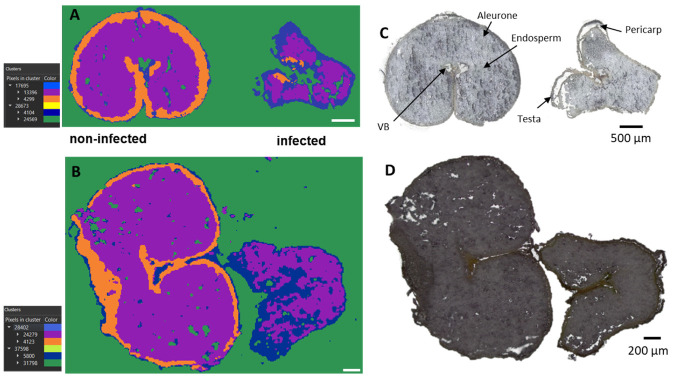
Bisecting k-means exploratory data analysis for AP-SMALDI imaging of durum (**A**) and common wheat (**B**) samples to visualize inter- and intra-sample comparison. (**C**,**D**) Optical images of (**C**) durum and (**D**) common wheat cross-sections. Segmentation clusters expanded to the third layer. Out-of-tissue (green) and in-tissue (blue, purple, and orange) clusters were identified. The hierarchy relations between clusters are shown in the legend.

A simultaneous analysis of healthy (DON < limit of detection) and diseased (DON content > 2000 µg/Kg) wheat kernels was performed. Infected and non-infected kernels were mounted on the same glass slide to ensure the results to be comparable. The optical images (Figure 1C,D) make it possible to distinguish the two because the infected grain is shriveled and lighter in weight and is usually whitish [27]. The section used in this study contained seed coat (pericarp and testa), aleurone, endosperm, and vascular bundle (Vb) (Figure 1C,D).

The metabolites detected in the two *Triticum* species (common and durum wheat) in response to the infection are reported in Table S1, while those discussed throughout the manuscript are listed in Table 1.

### 2.1. Unsupervised Data Mining

At first, the exploratory unsupervised data mining, including the segmentation analysis, was performed, revealing different regions of interest (ROI) (Figure 1A,B).

The segmentation clusters, expanded to the third layers, discriminate not only out-of-tissue (green cluster) and in-tissue (blue, purple, and orange) signals but also nicely distinguish between infected and control kernels. Furthermore, the segmentation clusters for both common and durum wheat samples strongly correlate with the seed compartments, such as the endosperm (violet cluster), aleurone (orange cluster), and pericarp (blue cluster) (Figure 1A,B). From Figure 1, it can be seen that both durum (Figure 1A) and common (Figure 1B) wheat shared the same behavior, indicating a common response to the infection despite their different genetic background.

In particular, the orange ROI, corresponding to the signals originating from the aleurone layers, was almost exclusively present in the cross-sections of the non-infected kernels. This is consistent with previous studies reporting severe damage and destruction of the aleurone layers in *Fusarium culmorum* highly-colonized seeds [28]. Similar effects of the infection were observed in maize kernels colonized by *Fusarium verticillioides* [29]. Thus, the damage to the seed coat and the aleurone layers is indeed due to the spread of the fungal hyphae, while the inner endosperm structure tends to remain intact. However, chemical changes in the endosperm layer were observed with the accumulation of linoleic acid, α-linoleic acid, and their metabolites, as displayed in Figures S1 and S2. In addition, elevated concentrations of the polyunsaturated free fatty acids linoleic and α-linolenic acid were previously detected in *F. graminearum*-infected wheat spike tissue, suggesting a role in establishing full *Fusarium* virulence [30].

Oxylipins deriving from the 13-lipoxygenase (13-LOX) pathway were found to be accumulated in the endosperm of infected common wheat kernels (Figures S1 and S2). Thus, the accumulation of 13-LOX pathway-related oxylipins in DON-contaminated wheat agrees with the literature since studies suggested that the 13-LOX pathway is activated after pathogen assault as a defense response in wheat [12] and corn [24,25,31].

The endosperm was also found to be the accumulation tissue for lysophosphatidylcholine (lysoPC), including lysoPC(16:0), lysoPC(18:0), lysoPC(18:2), lysoPC(18:1), and lysoPC(16:1), as shown in Figure S3. This tissue-dependent accumulation was previously reported in rice [17], with PC being localized in the bran and their lyso forms in the endosperm. The production of lysoPC and free fatty acids (i.e., linoleic acid and α-linoleic acid) is a consequence of the activation of phospholipase-mediated signaling pathways following pathogen recognition [32]. Phospholipase A (PLA) hydrolyzes membrane phospholipids to produce free fatty acids and lysophospholipids.

### 2.2. Localization of Signaling Molecules: Glycerolipids and Galactolipids

**Figure 2:**
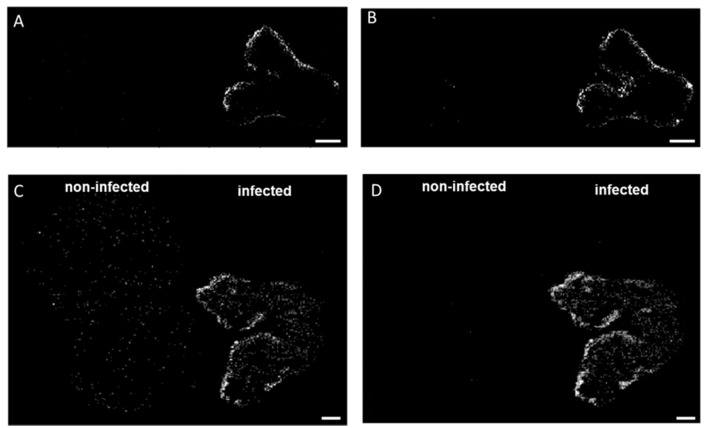
Spatial distribution of diacylglycerols in durum (**A**,**B**) and common wheat (**C**,**D**) cross-sections. (**A**,**C**) DG (33:4) [M + K]^+^ *m/z* 613.4228 and (**B**,**D**) DG (33:3) [M + K]^+^ *m/z* 615.4385 were found exclusively in the infected seeds. MS images were generated with (**A**,**B**) 336 × 138 pixels; 20 µm pixel size; *m/z* bin width: ±5 ppm; scale bars: 500 µm; (**C**,**D**) 300 × 220 pixels; 15 µm pixel size; *m/z* bin width: ±5 ppm; scale bars: 200 µm.

In addition to oxylipins and PCs, different lipid signaling molecules can be produced because of membrane modifications following fungal invasion, such as diacylglycerols (DG). These lipids result from the hydrolysis of phosphatidylinositol 4,5-bisphosphate operated by phospholipase C (PLC) [32,33]. Here, we detected DG (33:4) and DG (33:3) in the DON-contaminated common and durum wheat kernels. The latter DG was previously reported as a marker of DON-contaminated common wheat samples [12]. As shown in Figure 2, DGs were found to be localized in the outer layers of the seeds, which is in agreement with González-Thuillier et al., 2015 [34], who investigated the distribution of lipid species in common wheat using pearling fractioning. DGs were exclusively found in the contaminated samples, probably due to membrane alteration after a pathogen attack. Indeed, the activation of the enzyme responsible for the production of DGs (PLC) has been reported as essential for the growth and development of *Fusarium* spp. and having a possible role in trichothecene biosynthesis [35]. On the contrary, using a PLC-specific inhibitor results in a dose-dependent reduction in mycelial growth, conidiation, conidial germination, inhibition of perithecium, and colony formation [35].

**Figure 3:**
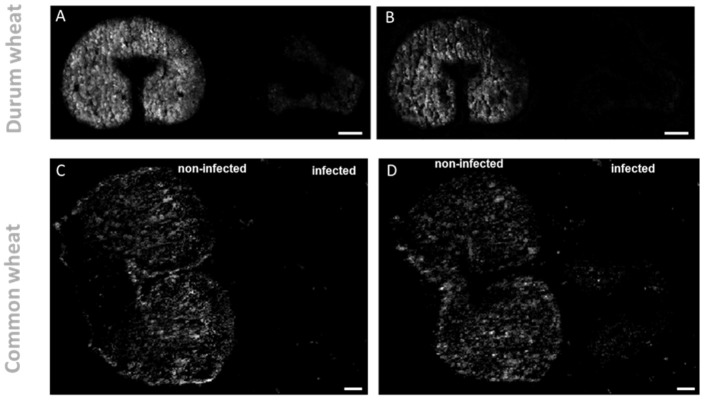
Spatial distribution of galactolipid in durum (**A**,**B**) and common wheat (**C**,**D**) cross-sections. (**A**,**C**). DGDG (36:4) [M + K]^+^ *m/z* 979.5735 and (**B**,**D**) MGDG(36:4) [M + K]^+^ *m/z* 817.5227 were found exclusively in the non-infected seeds. MS images were generated with (**A**,**B**) 336 × 138 pixels; 20 µm pixel size; *m/z* bin width: ±5 ppm; scale bars: 500 µm; (**C**,**D**) 300 × 220 pixels; 15 µm pixel size; *m/z* bin width: ±5 ppm; scale bars: 200 µm.

**Figure 4:**
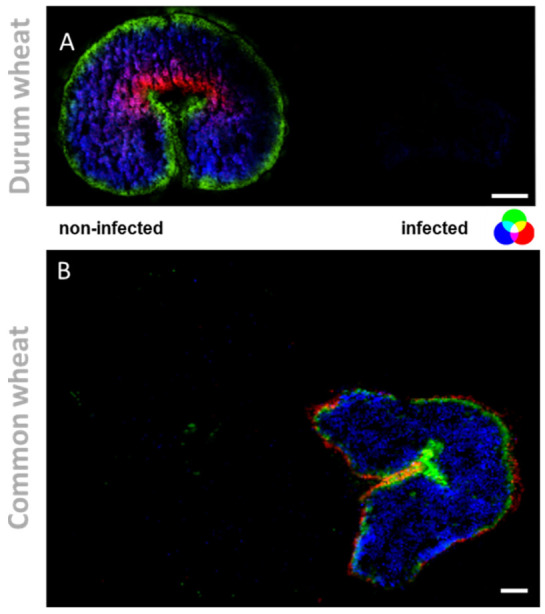
Tissue-specific changes, induced by mycotoxins accumulation in wheat kernels. (**A**) RGB overlay of *m/z* images of PE (37:1) *m/z* 798.5396 (red), DGDG (36:4) [M + K]^+^ *m/z* 979.5735 (blue) and MGDG (34:1) [M + K]^+^ *m/z* 795.383 (green). (**B**) RGB overlay of *m/z* images of coumaroylagmatine [M + H]^+^ *m/z* 277.1659 (red), linoleic acid [M + K]^+^ *m/z* 319.2033 (blue) and falcarindione [M + H]^+^ *m/z* 257.1536 (green). MS images were generated with (**A**) 336 × 138 pixels; 20 µm pixel size; *m/z* bin width: ±5 ppm; scale bars: 500 µm; (**B**) 300 × 220 pixels; 15 µm pixel size; *m/z* bin width: ±5 ppm; images normalized to the total ion count on a 0–60% intensity scale; scale bars: 200 µm.

On the other hand, the exclusive accumulation of DGs in infected kernels may also be used as building blocks for major galactolipids in chloroplasts and endoplasmic reticulum. Monogalactosyl-diacylglycerol (MGDG) (36:4) and digalactosyl-DG (36:4) showed the opposite trend as they were found to be solely accumulated in the non-infected kernels. As displayed in Figure 3 and Figure 4 these two lipids were found localized mainly in the endosperm tissue. This localization is consistent with previous reports [34,35,36], which indicate that MGDG and DGDG are the main polar lipids of wheat-seed endosperm included in the membrane of amyloplasts. DGDG (36:4) and MGDG (36:4) are reported to be the predominant species, accounting for 45−61% and 3−18%, respectively, of total galactolipids in common wheat [34]. The decrease of glycolipid contents has been previously reported for wheat and barley, following abiotic stressors, such as water stress [37]. On this account, it has been recently discovered that one of the main symptoms of *Fusarium* infection is the wilting of the entire plant, which could be caused by mycotoxins such as DON or a blockage of water transport [38,39].

Some *Fusarium* spp. have the ability to hydrolyze galactolipids by removing one or two fatty acids, due to the presence of enzymes named galactolipases [40], leading to the production of fungal oxylipins [41]. On the other hand, galactolipids may also be substrates for plant oxylipins production, since polyunsaturated fatty acids released from the galactolipids are preferred substrates for oxidation reactions catalyzed by lipoxygenases [42].

The activity of galactolipases will lead to the accumulation of galactolipid building-block, namely fatty acids and the disaccharide headgroup (galactopyranosyl-glucopyranosyl) that the plant will re-use for further biochemical reaction. As reported in Figure S4, we observed the accumulation of trisaccharides (galactopyranosyl-galactopyranosyl-arabinose), among other carbohydrates (see Table S1) in the endosperm of infected common and durum wheat kernels.

Higher abundances of sugars such as galactopyranose and myo-inositol derivatives are considered resistance-related metabolite-induced, following *Fusarium* [43] and not following only DON application. In addition, these signaling metabolites can regulate plant hormone receptors and participate in fungal recognition, mediating the plant wound responses [43,44,45].

### 2.3. Outer-Layers Localization of Secondary Antifungal Metabolites: Hydroxycinnamic Acid Amides

**Figure 5:**
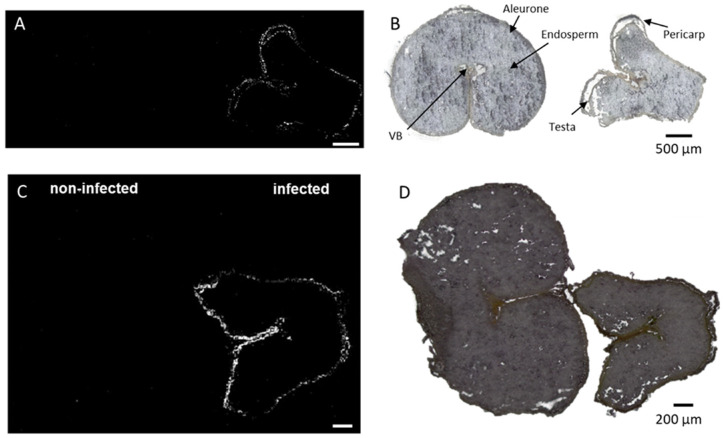
Spatial distribution of coumaroylagmatine in durum (**A**,**B**) and common wheat (**C**,**D**) cross-sections. Coumaroylagmatine [M + H]^+^ *m/z* 277.1659 was found exclusively in the outer cuticle of the pericarp in infected seeds. Optical image of durum (**B**) and common wheat (**D**) seed sections with major morphological features labeled. MS images were generated with (**A**) 336 × 138 pixels; 20 µm pixel size; *m/z* bin width: ±5 ppm; scale bars: 500 µm; (**B**) 300 × 220 pixels; 15 µm pixel size; *m/z* bin width: ±5 ppm; scale bars: 200 µm.

The fungal colonization progresses from the outer to the inner layer of the kernel. Therefore, reinforcing the outer protective layers is crucial for the plant to succeed in the plant−pathogen interaction. Here, a metabolite belonging to the phenylpropanoid pathway, coumaroylagmatine, was exclusively detected in infected wheat kernels (see Figure 4 and Figure 5 for the overlay). As highlighted in Figure 5, it was localized in the outer cuticle of the pericarp of infected kernels. Its localization in the outer layers of infected kernels is consistent with its biological activity as an antifungal compound [46], representing a passive defense against pathogens that colonize the rachis surface. The secretion of antimicrobial secondary metabolites is vital for the control of pathogen ingress. The rapid accumulation of hydroxycinnamic acid amides (HCAA), such as coumaroylagmatine, in response to DON-producing *Fusarium* strains, along with the described antimicrobial activity, indicates that the compounds can be classified as phytoalexins, playing a pivotal role in the plant response to pathogens [10]. Growing evidence also supports the role of this metabolite and other cinnamic acid derivatives in inhibiting the biosynthesis of mycotoxins, such as DON [11].The importance of HCAA metabolites is also due to their role as cell wall strengthening agents. One of the most accepted hypotheses is based on the ability of HCAA to bind to cell wall components by cross-linking to polysaccharides, resulting in a strengthening of the physical barrier that prevents or reduces fungal infection. Here, the specific tissue accumulation of coumaroylagmatine (see Figure 4 for the overlay) in the pericarp of infected kernels may suggest the reinforcement of the secondary cell walls of those cells and the reduction of further spreading of the pathogen from the infected site [10]. A similar speculation was reported by Negrel and co-authors [47] who localized ether-linked ferulate amides in the wound periderm of potato tubers, suggesting that the synthesis and integration of the compounds play a direct role in the early response to lesion and pathogen attack.

Co-localized in the outer bran with HCCA, we also identified a carnitine metabolite (see Figure S5). It was accumulated exclusively in the infected seed, according to previously reported data [21], co-localized with the *F. graminearum* hyphae in infected wheat seeds. Thus, L-carnitine and its metabolites may participate in protecting the plant cell against different types of stress through an action on the abscisic acid signaling pathway, but its role in plant physiology has been scarcely studied [48].

Another pathogenesis-related metabolite was found to be co-localized in the outer pericarp of infected wheat kernels, named ergosterol. As depicted in Figure S6, it was detected both as protonated and [M + H−H_2_O]^+^ adducts, and accumulated in the outer layers, consistent with the progress of the fungal colonization. The detection of ergosterol is considered an indicator for fungal invasion, being the predominant sterol in the fungal cell membrane. Its content significantly correlates with that of trichothecene [49]. However, DON was not spatially detected by AP-SMALDI MS imaging. As discussed in a previous study, the reason could be the low ionization efficiency of trichothecene mycotoxins by MALDI [50]. This distribution indicates a relationship with the pathogen infection since *Fusarium* spp. preferably colonizes this peripheral tissue in mature seeds.

## 3. Materials and Methods

### 3.1. Chemicals

The deoxynivalenol (DON) (10 mg L^−1^ in acetonitrile) was obtained from Romer Labs (Tulln, Austria). The 2,5-dihydroxybenzoic acid (DHB), trifluoroacetic acid (TFA), bidistilled water, MS-grade acetone, and acetonitrile were purchased from Sigma-Aldrich (Steinheim). The gelatin was obtained from VWR International (Darmstadt, Germany). The glass microscope slides (ground edges, super frost) were obtained from R. Langenbrinck (Emmendingen, Germany).

### 3.2. Plant Material

The control and contaminated grain seeds of common (*Triticum aestivum*) and durum wheat (*Triticum durum*) were randomly picked from the bulk of seeds. Wheat samples were previously checked for the presence of DON using the LC-MS (Liquid chromatography–mass spectrometry) technique.

### 3.3. Sample Preparation

The sample preparation for MALDI was performed following the protocol previously optimized [21]. To put it briefly, embedded samples in 2% gelatin were cut from the middle of each grain at −20 °C to obtain consecutive cryosections (20 µm thick). Due to the fragility of the seed, adhesive tape kept over the trimmed sample was used during cryosectioning (HM525 cryostat, Thermo Scientific, Dreiech, Germany). The sections on the glass slide were kept at −80 °C until the analysis day. Before matrix application, the sections’ optical images were captured by a digital microscope VHX-5000 (Keyence GmbH, Neu-Isenburg, Germany). DHB (30 mg mL^−1^) in acetone:water (50:50, *v/v*, 0.1% TFA) was sprayed with an ultrafine pneumatic sprayer (SMALDIPrep, TransMIT GmbH, Giessen, Germany) [51] to ensure uniform coating of tissue sections with the microcrystalline matrix. The size and uniformity of the deposited crystals were checked prior to AP-SMALDI MS imaging experiments. At least two biological replicates were analyzed by MSI.

### 3.4. AP-SMALDI MS Imaging Analysis

The wheat seed sections imaging experiments were acquired using a high-spatial-resolution (≥5 µm step size) atmospheric-pressure scanning microprobe matrix-assisted laser desorption/ionization MSI ion source (AP-SMALDI5 AF, TransMIT GmbH, Giessen, Germany) coupled to a Q Exactive HF orbital trapping mass spectrometer (Thermo Fisher Scientific GmbH, Bremen, Germany).

The minimum laser beam focus results in an ablation spot diameter of 5 μm [26,52]. For the experiments described below, laser step sizes pf at 15 and 20 μm, respectively, were set. The mass spectrometer was operated in positive-ion mode: scan range *m/z* 250–1000; spray voltage +3 kV; capillary temperature 250 °C, automatic gain control (AGC) disabled; cycle time 1.3 pixels/s. The internal mass calibration was performed using known matrix ion signals as lock mass values (*m/z* 716.12461), providing a mass accuracy of better than 2 ppm root mean square error over the entire measurement.

### 3.5. Data Processing and Image Generation

LIPOSTARMSI software [53] was employed for data processing and image generation, importing the raw data as imzML format.

The bisection k-means segmentation analyses [54] were applied to mine complex MSI datasets applying spatial denoising and total ion current (TIC) normalization [55,56]. The metabolite annotation of MSI data was performed against publicly available LIPID MAPS and PlantCyc databases by accurate *m/z* matching within user-set tolerances. The ion images of selected *m/z* values were generated with an *m/z* bin width of ±2 ppm and normalized to TIC. The raw data were deposited on the METASPACE platform (https://metaspace2020.eu, accession date 16 November 2021) [57].

## 4. Conclusions

Here, we reported the spatial distribution of plant defense metabolites involved in the fungal-plant cross-talk. Lipids and antifungal metabolites were finely located in infected vs. non-infected kernels thanks to the high-spatial resolution provided by (AP)-SMALDI MSI instrumentation. An important challenge for future research is to visualize the potential co-localization of major fungal metabolites (i.e., mycotoxins) with plant defense metabolites to decipher if the priming effect can act at a distance. Unfortunately, as mentioned above, the ionization of low *m/z* values mycotoxins by MSI is quite challenging, thus we could not visualize a reliable co-localization of DON and plant metabolites. Such a scenario suggests the importance of screening new MALDI matrices to improve the ionization efficiency and thus the metabolite coverage of a variety of natural toxins and small plant metabolites.

## Figures and Tables

**Table 1:** Differentially accumulated metabolites in infected vs. non-infected kernels, whose *m*/*z* images are reported in the manuscript (qualitative abundance are reported as: N.D., not detected; ++, detected with high relative abundance).

Class	Compounds	Localization	Infected Kernel	Non-Infected Kernel	Molecular Formula	Adduct	Exact Mass	Error ppm
Diacylglycerols	DG (33:4)	Pericarp	++	N.D.	C_36_H_62_O_5_	[M + K]^+^	613.4228	0.77
Diacylglycerols	DG (33:3)	Pericarp	++	N.D.	C_36_H_64_O_5_	[M + K]^+^	615.4385	0.64
Glycosyldiacylglycerols	DGDG (36:4)	Endosperm	N.D.	++	C_51_H_88_O_15_	[M + K]^+^	979.5755	0.18
Glycosyldiacylglycerols	MGDG (36:4)	Endosperm	N.D.	++	C_45_H_78_O_10_	[M + K]^+^	817.5227	0.24
Hydroxycinnamic acids amides	Coumaroylagmatine	Pericarp and testa	++	N.D.	C_14_H_20_N_4_O_2_	[M + H]^+^	277.1659	−0.31

Classes have been taken from LIPID MAPS and PlantCyc databases. MGDG: monogalactosyl-diacylglycerol; DGDG: digalactosyl-diacylglycerol.

## Data Availability

Mass spectrometry imaging data that support the findings of this study have been deposited in Metaspace (https://metaspace2020.eu/datasets, accessed date 16 November 2021) with the accession code JLU Giessen_ WS_combined_DHB_HF_336x138_20um_A15; WS_inf_DON_183x149_20_A15; T077 WS26 300x220 15um E65; S649 WS21 155x105 15um E110.

## Supplementary Materials

The following are available online at https://www.mdpi.com/article/10.3390/metabo12010048/s1, Table S1: List of metabolites imaged in common and durum wheat and differentially accumulated in non-infected vs. infected kernels, Figure S1: Spatial distribution of linoleic acid and oxylipins in common wheat cross sections, Figure S2: Spatial distribution of α-Linoleic acid in common wheat cross sections, Figure S3: Spatial distribution of lyso-phosphatidylcholine in durum and common wheat cross-sections, Figure S4: Spatial distribution of carbohydrates in durum and common wheat cross-sections, Figure S5: Spatial distribution of N-acyl amines in durum and common wheat cross-sections, Figure S6: Spatial distribution of ergosterol in durum wheat cross-sections.
