# Supplementary material for: Mass Spectrometry Imaging Disclosed Spatial Distribution of Defense-Related Metabolites in Triticum spp
Source: Metabolites. 2022 Jan 7;12(1):48. doi: 10.3390/metabo12010048 (PMC8780301; doi:10.3390/metabo12010048)
Supplement: Supplementary file 1 [file metabolites-12-00048-s001.zip › metabolites-1489666-supplementary.pdf]

Supplementary Information for

Mass Spectrometry Imaging Disclosed Spatial Distribution of Defense-Related Metabolites in *Triticum* spp.

Laura Righetti [1,2,*], Sven Gottwald [1], Sara Tortorella [3], Bernhard Spengler [1] and Dhaka Ram Bhandari [1,4,*]

[1] Institute of Inorganic and Analytical Chemistry, Justus Liebig University Giessen, Heinrich-Buff-Ring 17, 35392 Giessen, Germany; sv.gottwald@t-online.de (S.G.); bernhard.spengler@anorg.chemie.uni-giessen.de (B.S.)
[2] Food and Drug Department, University of Parma, Viale delle Scienze 17/A, 43124 Parma, Italy
[3] Molecular Horizon Srl., Via Montelino 30, Bettona, 06084 Perugia, Italy; sara@molhorizon.it
[4] Gandaki Prvince Academy of Science and Technology, Pokhara 33700, Nepal
* Correspondence: laura.righetti@unipr.it (L.R.); dhaka.r.bhandari@transmit.de (D.R.B.)

Table S1. List of metabolites imaged in common and durum wheat and differentially accumulated in non-infected vs infected kernels. The reference column gives cited references for metabolites detected in previous studies on wheat or other plants. The citation details are provided in the reference list.

**Subclass**	**Putative identification**	**Formula**	**Adduct**	**Detected *m/z***	**Infected kernel**	**Non-infected kernel**	**Ref**
N-acyl amines	Hydroxyhexadecadienoylcarnitine	$C_{23}H_{41}NO_5$	$[M+H]^+$	412.3057	Endosperm and aleurone	N.D.	[1]
	Hydroxy-hexadecenoylcarnitine	$C_{23}H_{43}NO_5$	$[M+H]^+$	414.3214	Endosperm and aleurone	N.D.	[1]
	Hexadecanedioic acid mono-carnitine ester	$C_{23}H_{43}NO_6$	$[M+H]^+$	430.3163	Endosperm and aleurone	N.D.	[1]
Unsaturated fatty acid	Alpha-Linolenic acid	$C_{18}H_{30}O_2$	$[M+H]^+$	279.2318	Endosperm	N.D.	[2]
	12,13-EOTrE	$C_{18}H_{28}O_3$	$[M+H]^+$	293.2111	Endosperm	N.D.	[3]
	Linoleic acid	$C_{18}H_{32}O_2$	$[M+K]^+$	319.2033	Endosperm	N.D.	[2]
	13-HOME	$C_{18}H_{34}O_3$	$[M+Na]^+$	321.2400	Endosperm	N.D.	[4]
	13-OxoODE	$C_{18}H_{30}O_3$	$[M+K]^+$	333.1826	Endosperm	N.D.	[3]
	13-HODE	$C_{18}H_{32}O_3$	$[M+K]^+$	335.1983	Endosperm	N.D.	[4]
	13-HpOTrE	$C_{18}H_{30}O_4$	$[M+K]^+$	349.1775	Endosperm	N.D.	[5]
	13-HpODE/ 12,13-DiHODE	$C_{18}H_{32}O_4$	$[M+K]^+$	351.1932	Endosperm	N.D.	[3]
	12,13-diHOME	$C_{18}H_{34}O_4$	$[M+K]^+$	353.2088	Endosperm	N.D.	[5]
	2-HpOTrE	$C_{18}H_{30}O_5$	$[M+K]^+$	365.1724	Endosperm	N.D.	
	TriHODE/ 9,10-Dihydroxy-8-oxo-12-octadecenoic acid	$C_{18}H_{32}O_5$	$[M+K]^+$	367.1881	Endosperm	N.D.	[5]
	TriHOME	$C_{18}H_{34}O_5$	$[M+K]^+$	369.2037	Endosperm	N.D.	[5]
Sterol	ergosterol	$C_{28}H_{44}O$	$[M+H-H_2O]^+$	379.3359	Endosperm	N.D.	[6]
			$[M+H]^+$	397.3465	Endosperm	N.D.	[6]
Vitamin D3 and derivatives	Hydroxy-oxo-hexanorvitamin D3	$C_{21}H_{30}O_3$	$[M+Na]^+$	353.2087	Endosperm	N.D.	[7]
	Dihydroxy-oxo-hexanorvitamin D3	$C_{21}H_{30}O_4$	$[M+Na]^+$	369.2036	Endosperm	N.D.	[7]
	Vitamin D3/7-Dehydrocholesterol	$C_{27}H_{44}O$	$[M+K]^+$	423.3023	Endosperm	N.D.	[7]
	Dihydroxy-epivitamin D3	$C_{27}H_{44}O_3$	$[M+K]^+$	455.2922	Endosperm	N.D.	[7]
	Trihydroxyvitamin D3	$C_{27}H_{44}O_4$	$[M+K]^+$	471.2871	Endosperm	N.D.	[7]

	Tetrahydroxyvitamin D3	$C_{27}H_{44}O_5$	$[M+K]^+$	487.2820	Endosperm	N.D.	[7]
	Dihydroxyl-didehydro-trihomovitamin D3	$C_{30}H_{48}O_3$	$[M+K]^+$	495.3235	Endosperm	N.D.	[7]
	Diethyl-dihydroxyl-didehydro-oxavitamin D3	$C_{30}H_{48}O_4$	$[M+K]^+$	511.3184	Endosperm	N.D.	[7]
Carbohydrates and carbohydrate conjugates	Tetra-hexose	$C_{24}H_{40}O_{20}$	$[M+K]^+$	687.1744	Endosperm	N.D.	[8]
	Apiosylglucosyl hydroxybenzoate	$C_{18}H_{24}O_{12}$	$[M+Na]^+$	455.1160	Endosperm	N.D.	[8]
Triterpenoids	Feruloylcorosocolic acid	$C_{40}H_{56}O_7$	$[M+H]^+$	649.4099	Pericarp and testa	N.D.	
	Gibbeellin glucosyl ester	$C_{25}H_{34}O_{11}$	$[M+H]^+$	511.2174	Pericarp and testa	N.D.	
	Methyl-apocarotenoate	$C_{33}H_{44}O_2$	$[M+Na]^+$	495.3233	Endosperm and aleurone	N.D.	
Phenolic glycosides	Flavonoid glycoside	$C_{34}H_{40}O_{22}$	$[M+Na]^+$	823.1903	Endosperm	N.D.	[9]
	Kaempferol rhamnoside	$C_{35}H_{42}O_{20}$	$[M+K]^+$	821.1901	Pericarp	N.D.	
	Methyl liquiritugenin rhamnoside	$C_{22}H_{24}O_8$	$[M+H]^+$	417.1544	Pericarp	N.D.	
Quinone and hydroquinone lipids	Vit D2 hydroquinone	$C_{21}H_{26}O_2$	$[M+Na]^+$	333.1825	Endosperm	N.D.	
Purine 2'-deoxyribonucleosides	Hydroxy-deoxyguanosine	$C_{10}H_{13}N_5O_5$	$[M+K]^+$	322.0548	Endosperm	N.D.	[10]
Carbonyl compounds	Falcarindione	$C_{17}H_{20}O_2$	$[M+H]^+$	257.1536	Pericarp and VB	N.D.	[11]
Simple Glc series	GlcCer (d14:1/20:0(OH))	$C_{40}H_{77}NO_9$	$[M+K]^+$	754.5230	Endosperm	N.D.	[5]
Glycerophosphates	PA (42:8)	$C_{45}H_{73}O_8P$	$[M+K]^+$	811.4674	Pericarp	N.D.	
Diacylglycerols	DG (36:8)	$C_{39}H_{60}O_5$	$[M+Na]^+$	631.4333	Pericarp	N.D.	[4]
Glycerophospholipids	PC(24:2) /PE-NMe(36:2)	$C_{42}H_{80}NO_8P$	$[M+H]^+$	758.5694	N.D.	Endosperm	
			$[M+Na]^+$	780.5513	N.D.	Endosperm	
	PC (24:1)/PE-NMe(36:1)	$C_{42}H_{82}NO_8P$	$[M+H]^+$	760.5850	N.D.	Endosperm	
	PE (20:4/P-18:1)	$C_{43}H_{76}NO_7P$	$[M+Na]^+$	772.5251	N.D.	Endosperm	
	PC (32:0), PE(35:0), PE-NMe(34:0)	$C_{40}H_{80}NO_8P$	$[M+K]^+$	772.5253	N.D.	Endosperm	
	PC (26:4)/ PE-NMe(38:4)	$C_{44}H_{80}NO_8P$	$[M+H]^+$	782.5694	N.D.	Endosperm	
	PE (22:6/P-18:1)	$C_{45}H_{76}NO_7P$	$[M+Na]^+$	796.5251	N.D.	Aleurone and endosperm	
	PG (40:6)	$C_{46}H_{79}O_{10}P$	$[M+H]^+$	823.5448	N.D.	Aleurone	
	PG (36:2)	$C_{42}H_{79}O_{10}P$	$[M+Na]^+$	797.5303	N.D.	Endosperm	
	PE (22:5/P-18:1)	$C_{45}H_{78}NO_7P$	$[M+Na]^+$	798.5408	N.D.	Aleurone and endosperm	

	PS (46:7)	$C_{52}H_{88}NO_{10}P$	$[M+K]^+$	956.5777	N.D.	Endosperm	
	LysoPC (16:0)	$C_{24}H_{50}NO_7P$	$[M+Na]^+$	518.3217	N.D.	Endosperm	
Glycosylglycerols	MGDG (34:2)	$C_{43}H_{78}O_{10}$	$[M+K]^+$	793.5226	N.D.	Endosperm	[12]
	DGDG (36:9)	$C_{51}H_{78}O_{15}$	$[M+K]^+$	795.5383	N.D.	Endosperm	[12]
	MGDG (36:9)	$C_{45}H_{68}O_{10}$	$[M+K]^+$	796.5410	N.D.	Endosperm	[12]
	Glycerol -(octadecadienoate) -hexadecanoate-[galactopyranosyl-galactopyranoside]	$C_{49}H_{88}O_{15}$	$[M+Na]^+$	939.6015	N.D.	Endosperm	

- **Linoleic acid metabolism**

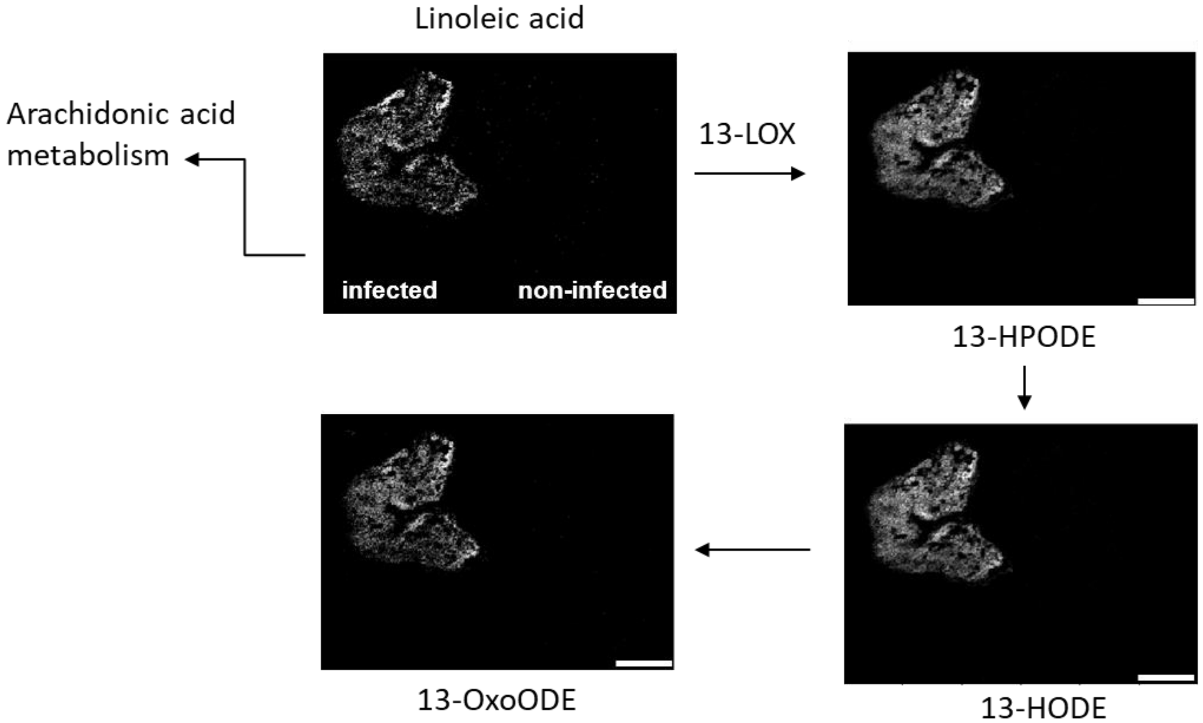

Figure S1. Spatial distribution of linoleic acid and oxylipins in common-wheat cross-sections: linoleic acid $[M+K]^+$ *m/z* 319.2034, 13-HPODE $[M+K]^+$ *m/z* 351.1932, 13-HODE $[M+K]^+$ *m/z* 335.1983, 13-oxoODE $[M+K]^+$ *m/z* 333.1826.
MS images were generated with 300 x 220 pixels; 15 µm pixel size; *m/z* bin width: ± 5 ppm; Scale bars: 400 µm.

- **α-Linoleic acid metabolism**

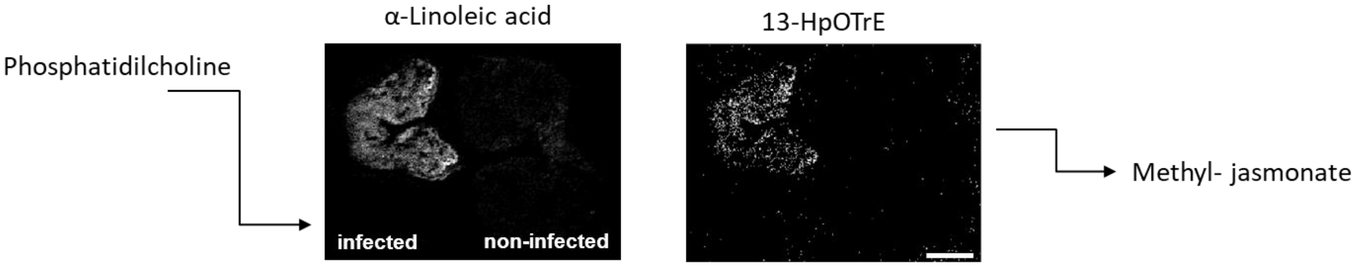

Figure S2. Spatial distribution of α-linoleic acid $[M+H]^+$ *m/z* 279.2318 and 13-HpOTrE $[M+K]^+$ *m/z* 349.1775 in common-wheat cross-sections.
MS images were generated with 300 x 220 pixels; 15 µm pixel size; *m/z* bin width: ± 5 ppm;. Scale bars: 400 µm.

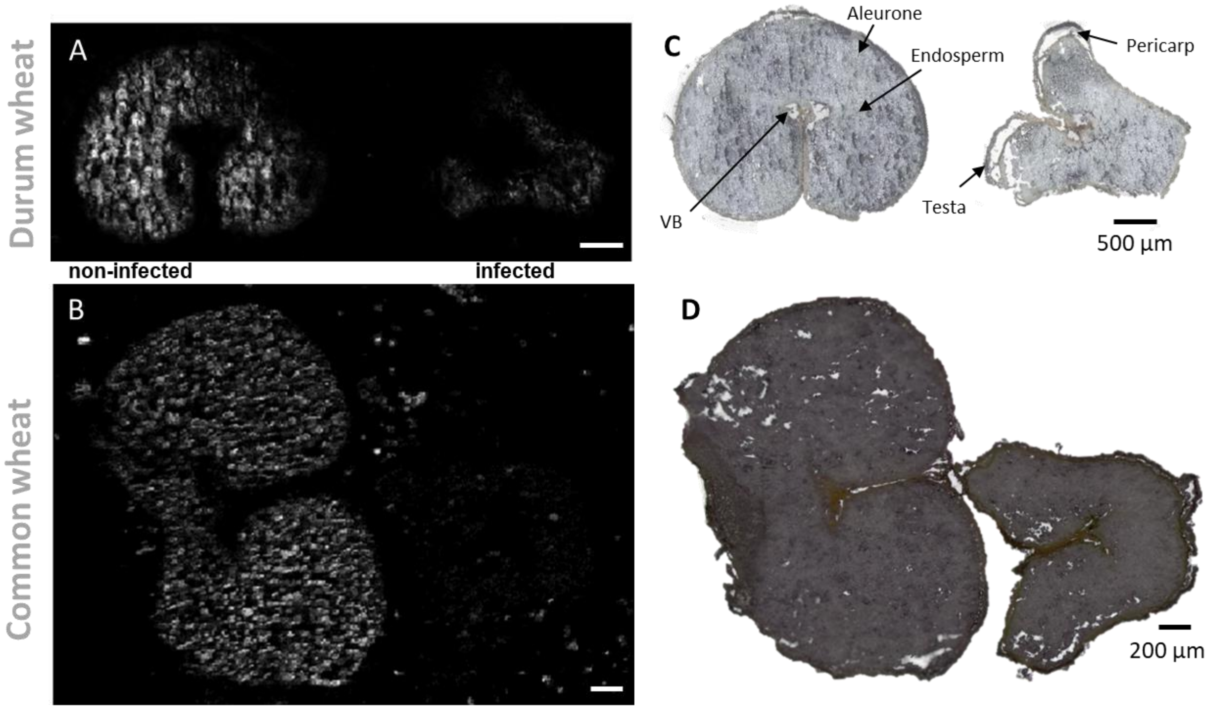

Figure S3. Spatial distribution of lyso-phosphatidylcholine in durum- (A) and common-wheat (B) cross-sections. LysoPC (16:0) $[M+Na]^+$ *m/z* 518.3216 was found accumulated mainly in the endosperm of the non-infected seed. Optical image of durum- (C) and common-wheat (D) seed section with major morphological features labeled.

MS images were generated with (A) 336 x 138 pixels; 20 µm pixel size; *m/z* bin width: ± 5 ppm; scale bars: 500 µm; (B) 300 x 220 pixels; 15 µm pixel size; *m/z* bin width: ± 5 ppm; scale bars: 200 µm.

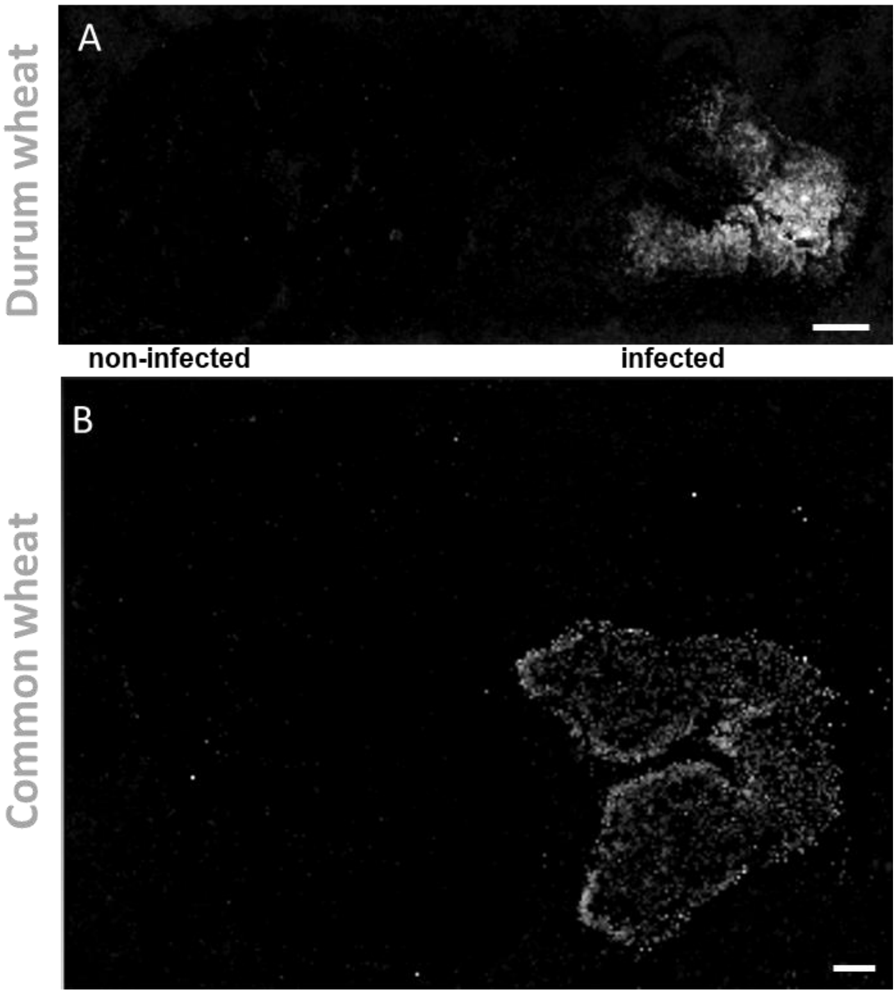

Figure S4. Spatial distribution of carbohydrates in durum- (A) and common-wheat (B) cross-sections. Trisaccharide $[M+K]^+$ *m/z* 513.1222 was found accumulated exclusively in the infected seed.
MS images were generated with (A) 336 x 138 pixels; 20 µm pixel size; *m/z* bin width: ± 5 ppm; scale bars: 500 µm; (B) 300 x 220 pixels; 15 µm pixel size; *m/z* bin width: ± 5 ppm; scale bars: 200 µm.

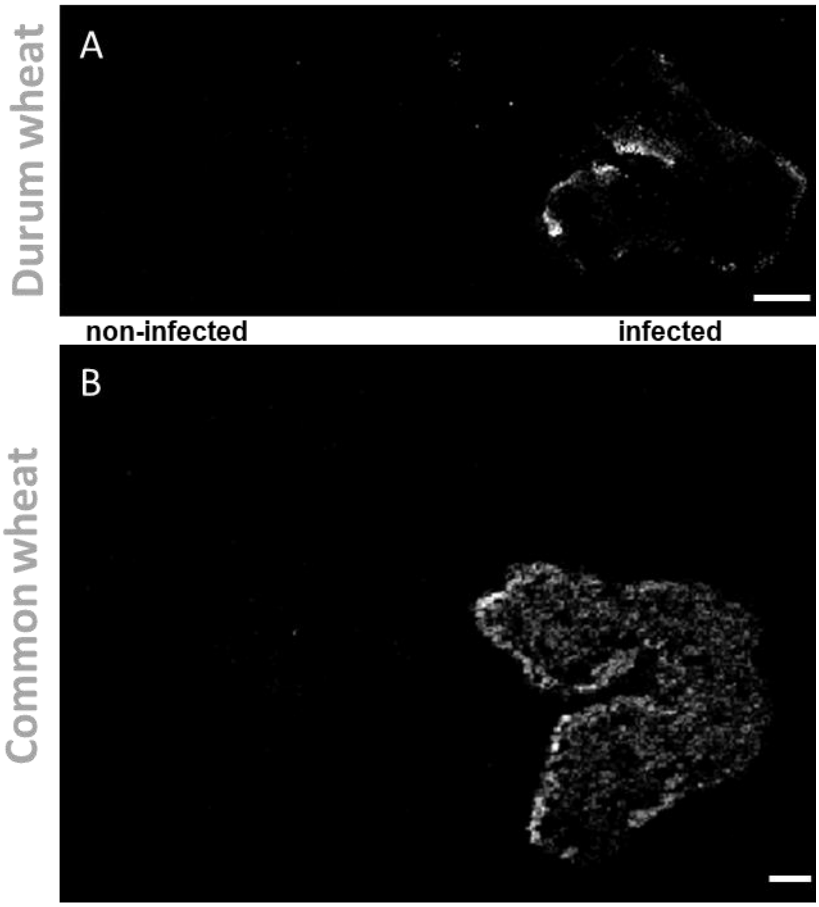

Figure S5. Spatial distribution of N-acyl amines in durum- (A) and common-wheat (B) cross-sections. Hydroxy-hexadecadienoylcarnitine $[M+H]^+$ *m/z* 412.3057 was found accumulated exclusively in the infected seed.

MS images were generated with (A) 336 x 138 pixels; 20 µm pixel size; *m/z* bin width: ± 5 ppm; scale bars: 500 µm; (B) 300 x 220 pixels; 15 µm pixel size; *m/z* bin width: ± 5 ppm; scale bars: 200 µm.

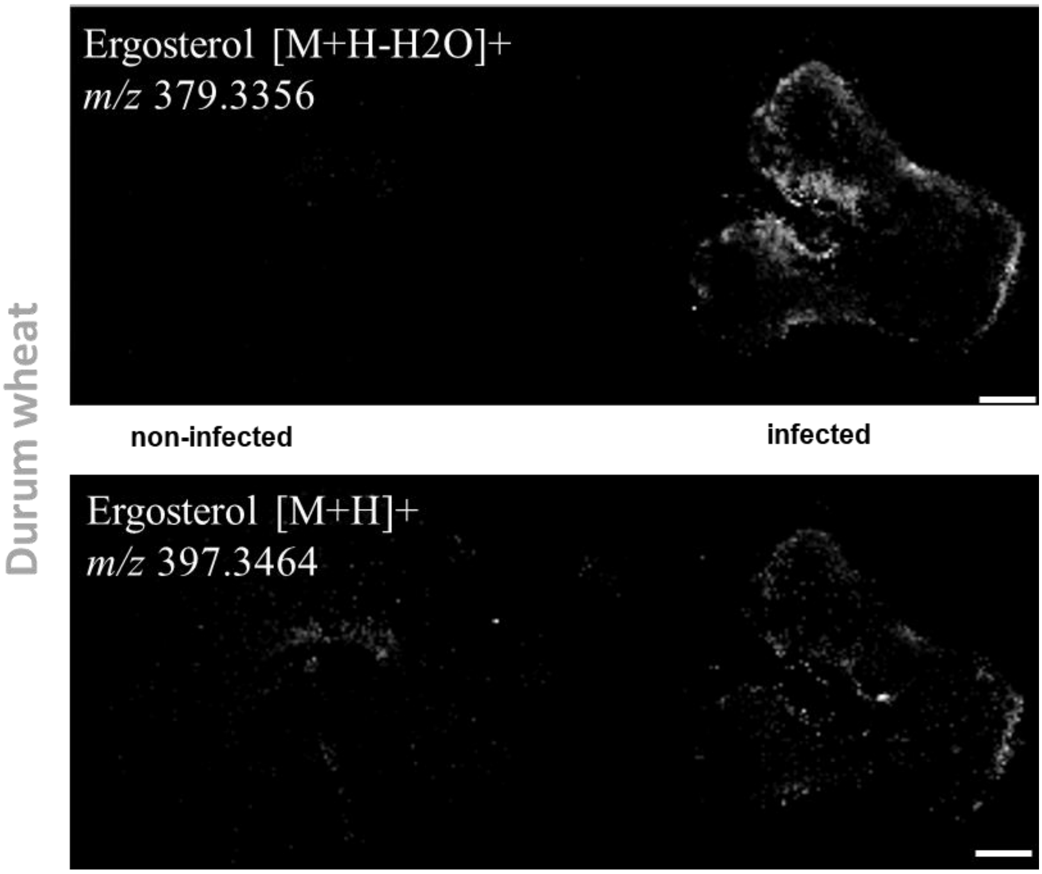

Figure S6. Spatial distribution of ergosterol in durum-wheat cross-sections. The $[M+H]^+$ image was normalized to the total ion count on a 0-60% intensity scale.
MS images were generated with 336 x 138 pixels; 20 µm pixel size; *m/z* bin width: ± 5 ppm; scale bars: 500 µm.

References

1. Bhandari, D.R.; Wang, Q.; Friedt, W.; Spengler, B.; Gottwald, S.; Römpp, A. High resolution mass spectrometry imaging of plant tissues: towards a plant metabolite atlas. *Analyst* **2015**, *140*, 7696–7709, doi:10.1039/C5AN01065A.

2. Righetti, L.; Lucini, L.; Giorni, P.; Locatelli, S.; Dall'Asta, C.; Battilani, P. Lipids as Key Markers in Maize Response to Fumonisin Accumulation. *J. Agric. Food Chem.* **2019**, *67*, 4064–4070, doi:10.1021/acs.jafc.8b06316.

3. Tsitsigiannis, D.I.; Keller, N.P. Oxylipins as developmental and host-fungal communication signals. *Trends Microbiol.* 2007, *15*.

4. Rubert, J.; Righetti, L.; Stranska-Zachariasova, M.; Dzuman, Z.; Chrpova, J.; Dall'Asta, C.; Hajslova, J. Untargeted metabolomics based on ultra-high-performance liquid chromatography–high-resolution mass spectrometry merged with chemometrics: A new predictable tool for an early detection of mycotoxins. *Food Chem.* **2017**, *224*, 423–431, doi:10.1016/j.foodchem.2016.11.132.

5. Scala, V.; Giorni, P.; Cirlini, M.; Ludovici, M.; Visentin, I.; Cardinale, F.; Fabbri, A.A.; Fanelli, C.; Reverberi, M.; Battilani, P.; et al. LDS1-produced oxylipins are negative regulators of growth, conidiation and fumonisin synthesis in the fungal maize pathogen Fusarium verticillioides. *Front. Microbiol.* **2014**, *5*, doi:10.3389/fmicb.2014.00669.

6. Varga, M.; Bartók, T.; Mesterházy, Á. Determination of ergosterol in Fusarium-infected wheat by liquid chromatography-atmospheric pressure photoionization mass spectrometry. *J. Chromatogr. A* **2006**, *1103*, doi:10.1016/j.chroma.2005.11.051.

7. Jäpelt, R.B.; Jakobsen, J. Vitamin D in plants: A review of occurrence, analysis, and biosynthesis. *Front. Plant Sci.* 2013, *4*.

8. Gauthier, L.; Atanasova-Penichon, V.; Chéreau, S.; Richard-Forget, F. Metabolomics to Decipher the Chemical Defense of Cereals against Fusarium graminearum and Deoxynivalenol Accumulation. *OPEN ACCESS Int. J. Mol. Sci* **2015**, *16*, 16, doi:10.3390/ijms161024839.

9. Bhandari, D.R.; Wang, Q.; Li, B.; Friedt, W.; Römpp, A.; Spengler, B.; Gottwald, S. Histology-guided high-resolution AP-SMALDI mass spectrometry imaging of wheat-Fusarium graminearum interaction at the root–shoot junction. *Plant Methods* **2018**, *14*, 103, doi:10.1186/s13007-018-0368-6.

10. Floyd, R.A.; West, M.S.; Hogsett, W.E.; Tingey, D.T. Increased 8-Hydroxyguanine Content of Chloroplast DNA from Ozone-Treated Plants. *Plant Physiol.* **1989**, *91*, doi:10.1104/pp.91.2.644.

11. Perochon, A.; Benbow, H.R.; Ślęczka-Brady, K.; Malla, K.B.; Doohan, F.M. Analysis of the chromosomal clustering of Fusarium-responsive wheat genes uncovers new players in the defence against head blight disease. *Sci. Rep.* **2021**, *11*, doi:10.1038/s41598-021-86362-4.

12. Chetal, S.; Wagle, D.S.; Nainawatee, H.S. Glycolipid changes in wheat and barley chloroplast under water stress. *Plant Sci. Lett.* **1981**, *20*, doi:10.1016/0304-4211(81)90266-2.
